# Cell Surface-Associated Anti-MUC1-Derived Signal Peptide Antibodies: Implications for Cancer Diagnostics and Therapy

**DOI:** 10.1371/journal.pone.0085400

**Published:** 2014-01-08

**Authors:** Riva Kovjazin, Galit Horn, Nechama I. Smorodinsky, Michael Y. Shapira, Lior Carmon

**Affiliations:** 1 Vaxil BioTherapeutics Ltd., Weizmann Science Park, Nes-Ziona, Israel; 2 Department of Cell Research and Immunology, George S. Wise Faculty of Life Sciences, Tel Aviv University, Tel-Aviv, Israel; 3 The Alec and Myra Marmot Hybridoma Unit, George S. Wise Faculty of Life Sciences, Tel Aviv University, Tel-Aviv, Israel; 4 Department of Bone Marrow Transplantation and Cancer Immunotherapy, Hadassah-Hebrew University Medical Center, Jerusalem, Israel; Technical University of Braunschweig, Germany

## Abstract

The MUC1 tumor associated antigen is highly expressed on a range of tumors. Its broad distribution on primary tumors and metastases renders it an attractive target for immunotherapy. After synthesis MUC1 is cleaved, yielding a large soluble extracellular alpha subunit containing the tandem repeats array (TRA) domain specifically bound, via non-covalent interaction, to a smaller beta subunit containing the transmembrane and cytoplasmic domains. Thus far, inconclusive efficacy has been reported for anti-MUC1 antibodies directed against the soluble alpha subunit. Targeting the cell bound beta subunit, may bypass limitations posed by circulating TRA domains. MUC1’s signal peptide (SP) domain promiscuously binds multiple MHC class II and Class I alleles, which upon vaccination, generated robust T-cell immunity against MUC1-positive tumors. This is a first demonstration of non-MHC associated, MUC1 specific, cell surfaces presence for MUC1 SP domain. Polyclonal and monoclonal antibodies generated against MUC1 SP domain specifically bind a large variety of MUC1-positive human solid and haematological tumor cell lines; MUC1-positive bone marrow derived plasma cells obtained from multiple myeloma (MM)-patients, but not MUC1 negative tumors cells, and normal naive primary blood and epithelial cells. Membranal MUC1 SP appears mainly as an independent entity but also co-localized with the full MUC1 molecule. MUC1-SP specific binding in BM-derived plasma cells can assist in selecting patients to be treated with anti-MUC1 SP therapeutic vaccine, ImMucin. A therapeutic potential of the anti-MUC1 SP antibodies was suggested by their ability to support of complement-mediated lysis of MUC1-positive tumor cells but not MUC1 negative tumor cells and normal naive primary epithelial cells. These findings suggest a novel cell surface presence of MUC1 SP domain, a potential therapeutic benefit for anti-MUC1 SP antibodies in MUC1-positive tumors and a selection tool for MM patients to be treated with the anti-MUC1 SP vaccine, ImMucin.

## Introduction

MUC1 is a mucin-like glycoprotein highly expressed on a range of epithelial carcinomas, including lung, breast, ovary, prostate and colon, as well as on the surface of haematological tumors, such as multiple myeloma (MM) [1], [2], [3], [4], [5], [6]. Its broad distribution on both primary tumor and metastasis, including cancer stem cells [7], has established it as a widely explored target for immunotherapy [1], [8], [9], [10]. In fact, MUC1 was listed by the National Cancer Institute pilot project as the second most promising target from a list of 75 potential tumor associated antigens (TAA) [11].

MUC1 exists in a number of isoforms [12], where the most extensively studied form is the polymorphic type I transmembrane protein (MUC1-TM), consisting of an extracellular domain containing 20–125 20-amino acid-long tandem repeat arrays (TRA) followed by a transmembrane domain and a short cytoplasmic tail [13], [14]. MUC1 is processed in the secretory pathway, yielding a large extracellular alpha subunit containing the TRA domain, non-covalently bound to a smaller beta subunit containing the molecule's transmembrane and cytoplasmic domains [15]. To date, while most anti-MUC1 antibodies target the TRA domain of the extracellular alpha subunit [16], [17], [18], studies have shown conflicting results regarding the immunotherapeutic efficacy of such antibody-based TRA–epitope targeting [19], [20], [21], [22], [23], [24], [25], [26]. These inconsistent findings are proposed to be the consequence of the non-covalent linkage of the TRA domain to the tumor cell surface; the soluble, circulating form acts as a decoy for anti-TRA antibodies, limiting their ability to reach MUC1-expressing tumor cells [23], [25]. Consequently, targeting MUC1 noncirculating epitopes exclusively expressed on tumor cell surfaces could potentially bypass these limitations. For this purpose, epitopes from the extracellular and intracellular segments surrounding the MUC1 TRA domain, along with epitopes within MUC1's signal peptide (SP) domain, were identified [20], [21], [27], [28].

SPs are short 13–50 amino acid-long lipophilic sequences typically located at the amino-terminus of proteins destined for secretion or for integration within cellular membranes [29]. Once protein translation is completed, SPs incorporated in the endoplasmic reticulum (ER) membrane are generally removed from the mature protein, but can still enter the ER lumen and bind MHC molecules, either directly, due to the unique protease activity of ER-membrane-associated signal peptide peptidase (SPP) [29], or indirectly, like other degraded sequences, via the transporter associated with antigen processing (TAP) machinery [30]. Yet, ER localization and MHC binding proficiency of SPs [31] relies both on their hydrophobic nature and specific sequence. Namely, alongside maintenance of the consensus motif required as a targeting signal, different SPs exhibit high variability and antigen specificity [29], [32], [33]. Consequently, SP domains can serve as vaccine candidates (VCs), inducing antigen-specific immune responses in a large portion of the population.

The 21-mer SP domain of MUC1 (MUC1 SP), herein the MUC1-SP-L or VXL100 peptide or the formulated therapeutic vaccine, ImMucin [28], is processed and presented in association with multiple MHC class I and II on the cell surface of both antigen presenting cells and various MUC1-positive tumor cells, which can generate robust T-cell immunity against MUC1-positive tumors [28]. In addition, a MUC1-specific humoral response can be generated against MUC1 SP, as manifested by significant elevation of natural autoantibodies in the bloodstream of MM patients but not in healthy donors [34]. Since soluble MUC1 SP was not detected in patient sera [34], it was speculated that the naturally generated autoantibodies were primed by non-MHC-restricted, MUC1-associated tumor cell-bound SP. The current study explored this possibility by generating specific polyclonal and monoclonal antibodies to MUC1-SP-L. Cell-surface presence of MUC1 SP was detected, using the raised antibodies, on tumor cell-lines and primary tumors, but not on naïve primary cells. In addition to their direct anti-tumor therapeutic potential, these antibodies can improve the selection criteria of MM patients aimed to be treated with the ImMucin anti-MUC1 SP therapeutic vaccine.

## Materials and Methods

### Peptide Synthesis

MUC1-SP-L, MUC1-SP-M, MUC1-SP-S1, MUC1-SP-S2, MUC1-SP-S3, MUC1-SP-S4, MUC1-SP-S5 and TB-Rv0476/4941-SP-L were synthesized by fully automated, solid-phase, peptide synthesis using fluorenylmethyloxycarbonyl (Fmoc)/tBu-strategy and Rink-amide-polystyrene resin (EMC Microcollections, Germany and ALMAC Sciences, UK). MUC1-TRA-L and BAGE-SP-L were synthesized using the same methodology (GL Biochem, China). Peptide purity and identity was >95%, as determined by high performance liquid chromatography and mass spectrometry analyses.

### Tumor Cell-lines and Hybridomas

The human B-lymphocytic leukemia lines Raji and Ramos, the human MM cell lines U266, and RPMI8226 and the human PC leukemia line ARH-77 were grown in suspension in RPMI-1640 medium supplemented with 10% fetal bovine serum (FBS), 2 mM L-glutamine, 1 mM sodium pyruvate, 1% non-essential amino-acid, 1 mM HEPES and 50 ug/ml gentamycin. The human ovarian carcinoma line OVARCAR-3 was grown as an adherent monolayer in RPMI-1640 medium supplemented with 20% FBS, 2 mM L-glutamine, 1 mM sodium pyruvate and 50 ug/ml gentamycin. The human ovarian carcinoma line ES-2, melanoma line SK-mel-28, and the breast cancer cell lines MCF7, MDA-231 and MDA-453 were grown as adherent monolayers and the human melanoma line SK-mel-1 was grown in suspension in DMEM, supplemented with 10% FBS, 2 mM L-glutamine, 1 mM sodium pyruvate and 50 ug/ml gentamycin. All cell lines were purchased from ATCC (Manassas VA, USA). All hybridomas used in this study were grown in DMEM supplemented with 10% horse serum, 2 mM L-glutamine, 1 mM sodium pyruvate and 50 ug/ml gentamycin. All culture reagents were purchased from Biological Industries, (Bet-Haemek, Israel).

### Antibodies

The anti-MUC1 TRA mAb H23, raised against the human breast cancer cell line T47D, [35] recognizing the non-glycosylated MUC1 epitope APDTRP, served as a positive control. Mouse anti-goat or rabbit anti-mouse IgG-FITC (Jackson ImmunoResearch, USA) served as a negative control for FACS analyses. Normal mouse or rabbit IgG antibodies (Chemicon, Millipore, USA) were used for complement-dependent cytotoxicity (CDC) analyses.

### Animals

Eight-week-old female BALB/c mice (Tel Aviv University breeding facility) and two-month-old rabbits (Harlan, Jerusalem, Israel) were maintained in the university animal research facility.

All experimental procedures involving mice and rabbits were approved by the Tel Aviv University Animal Care Committee.

### Patient Bone Marrow (BM) Aspirates and Primary Naïve Healthy Cells

BM aspirates (2–3 ml) were drawn from four patients (ages 50–75) with slowly progressing, asymptomatic MM, who had been screened for enrolment into ImMucin's phase I/II clinical trial (protocol VAXIL-001). The study was approved by the Ethics Committee of Hadassah University Hospital, Jerusalem, Israel the Israeli Ministry of Health and was registered at the PRS as NCT01232712. The analysis of BM-derived PC was performed within the framework of the screening process of the study and written informed consent from the MM patients was obtained for using their sample for research. Fresh normal human BM (Cat. No. 1 M-105) and human NHEC (Cat. No. CC-2251), were purchased from Lonza BioResearch (Somerset, NJ, USA). Normal human white blood cells were isolated from buffy-coat samples donated by the Israeli National Blood Bank, from 3 naïve donors.

### Production of Anti-MUC1 SP Polyclonal Antibodies

Four rabbits (R22, R23, R32 and R33) were subcutaneously immunized four times, at two-week intervals, with 500 ug keyhole limpet hemocyanin (KLH)-conjugated MUC1-SP-M, emulsified in complete Freund’s adjuvant in the first immunization and in incomplete Freund’s adjuvant in subsequent immunizations. KLH-peptide conjugation was performed by Adar Biotech (Rehovot, Israel) by means of glutaraldehyde-driven crosslinking. Seven days after the final immunization, rabbit sera were examined for the presence of MUC1-SP-M-specific antibodies; positive sera (titer 1∶12,800) were collected and pooled. IgG fractions of rabbit R23, termed R23IgG, used for all immunological assays, underwent ammonium sulfate (40%) precipitation before use, as previously described [36].

### Production of Anti-MUC1 SP mAbs

Four BALB/c mice were subcutaneously immunized as described above. Two weeks after the final immunization, mice sera were assessed for the presence of MUC1-SP-M-specific antibodies. Spleen cells from the highest positive sera-bearing mouse were harvested and fused with the murine NSO myeloma partner cell line, using polyethylene glycol (molecular weight 1500) (Roche Diagnostics GmbH, Germany). Hybridomas were selected for 2 weeks in hybridoma growth medium supplemented with 2% hypoxanthine-aminopterin-thymidine and were further cultured in hybridoma growth medium supplemented with 2% hypoxanthine-thymidine. ELISA was performed to screen culture supernatants for the presence of anti-MUC1-SP-M IgG Abs. Positive samples were rescreened by ELISA and were further subcloned and expanded. Large-scale Ab production of selected clones was achieved by purifying mAbs using an anti-mouse IgG agarose column (Sigma, Israel; cat. no A6531). Isotyping of mAbs was performed using an IsoStrip kit (Roche; cat. no. 1493027).

### ELISA-based Screening of Mouse Hyperimmune Sera and of Anti-MUC1-SP-M IgG-producing Hybridomas

ELISA plates (F96 Maxisorp, Nunc, Denmark) were activated for 1 h with 0.1% glutaraldehyde (Sigma, Israel) in carbonate buffer, pH = 9. Next, plates were coated with 50 ul peptide (5 ug/ml), in carbonate buffer (overnight, 4°C), followed by blocking (2 h, room temperature) with PBS supplemented with 5% FBS and 0.04% Tween 20 (ICN Biomedical Inc, USA). Sera samples from MUC1-SP-M-immunized mice were then diluted in the blocking buffer, while samples from hybridoma cultures were not diluted. All samples were incubated (2 h, room temperature), before extensive washing and treatment (1 h, room temperature) with HRP-conjugated anti-mouse IgG (Jackson ImmunoResearch, USA; 50 ul/well), following a 1∶10,000 dilution in blocking buffer. After extensive washing, plates were developed with TMB/E solution (CHEMICON, Millipore, USA), according to the manufacturer's instructions.

For peptide antibody competition assays, hyperimmune sera and hybridoma growth medium were incubated with 1 ug/well peptides in 96-well ELISA plates (F96 Maxisorp, Nunc, Denmark) activated with glutaraldehyde. The rest of the assay was performed as described above. A decrease of at least 50% in the OD was considered a positive result.

### Flow Cytometry and Imagestream

To confirm surface staining of MUC1 on tumor cell lines and co-expression of MM markers and MUC1 on BM aspirates, cells were washed and incubated for 30 min in staining buffer, consisting of PBS, supplemented with 3% FBS, 10% human AB sera and 0.1% sodium azide. BM “large cells” were initially gated by side vs. forward scatter. Next, cells were stained with one or more of the following: anti-human CD138-APC commercially conjugated Abs (IQ Products, Netherlands), anti-human kappa light chain-eFluor 450 and anti-human lambda light chain-PE (eBioscience, USA) and/or in-house-prepared FITC- or PE-conjugated anti-human MUC1 (anti-MUC1-TRA H23), anti-MUC1 SP R23IgG, SPmAb-6 and SPmAb-2.1 antibodies. FITC and PE conjugation was performed according to the manufacturer's protocol (Lighting link R-Phycoerythrin Conjugation Kit, Innova Biosciences, USA). Labeled cells were washed twice, fixed in BD CellFIX (Becton Dickenson, USA), according to the manufacturer's protocol, and stored at 4°C until analysis. At least 1×10^6^, and 3×10^4^ events were acquired for flow cytometry and image stream, respectively. For imagestream analysis, cell nuclei were stained with Hoechst staining solution (3030145), according to the manufacturer's protocol (MP Biomedicals, CA, USA). Flow cytometry analysis was performed with the LSR II (Becton Dickenson Immunocytometry Systems, USA) and data were analyzed using FlowJo software (TreeStar, USA). Colocalization analysis was performed with the Imagestream system (ImageStreamX flow cytometer; Amnis Corp) and analyzed using the IDEAS similarity Bright Detail feature (IDEAL 6.0; Amnis Corp). The similarity score is a measure of the degree to which two images are linearly correlated.

### Immunofluorescence Microscopy

Adherent cells (5×104 cells/well) were plated onto glass coverslips (Marlenfeld GmbH & Co), in 24-well plates, for 18 h 37°C. Glass coverslips were washed twice with cold (4°C) PBS, fixed with 4% paraformaldehyde (20 min, room temperature), blocked and permeabilized with PBS, supplemented with 3% BSA and 0.1% triton (1 h, room temperature). Next, cells were stained (1 h, room temperature) with antibodies diluted to 20 ug/ml in staining solution (1% BSA, 0.1% triton in PBS), and then with secondary antibody, diluted 1∶200, in staining solution supplemented with DAPI (30 min, room temperature). Slides were mounted with Fluorescence Mounting medium (Golden Bridge Life Science, USA). Cells grown in suspension were stained with primary and secondary antibodies and then fixated and plated on glass coverslips. Cells were viewed with a Zeiss 100×, NA 1.4, Yokogawa CSU-22, or Zeiss fully–automated-inverted 200 M microscope, with solid state lasers 473, 561 and 660 nm; piezo-controlled Z-stage all under the command of Slidebook™. Images were acquired with an Evolve EMCCD camera (Photometrics; 100×lens, 1×1 binning, pixel size: 0.16 microns).

### CDC

Various tumor lines and HMEC (Target cells) (1×10^6^ cells/ml) were labelled with 2 uCi/ml 3[H]-thymidine (Amersham, UK), for 18 h at 37°C. Next, cells were washed three times with PBS and incubated (2 h, room temperature) with 100, 50 or 10 ug/ml H23, R23IgG, SPmAb-6 or SPmAb-2.1 antibodies. Cells were then washed with PBS, and 1×10^4^ cells were incubated (5 h, 37°C) in 4 ml tubes with 20 ul, 10 ul or 5 ul human serum complement (Sigma, Israel). Cells were then washed three times with PBS, resuspended in 300 ul PBS and seeded in 100 ul/well triplicates, in 96-well plates (Griner, De Groot, Germany). Cell harvesting was performed using unifilter 96-well plates (PerkinElmer, USA). Radioactivity was determined in a beta-counter (PerkinElmer, IL, USA). For spontaneous lysis, cells were incubated under the same conditions, but without addition of complement. For total lysis, 10% triton X100 was added to the labelled cells. Percentage of specific lysis was calculated as follows: (CPM in experimental well – CPM in spontaneous sample)/(CPM in total sample – CPM in spontaneous sample)×100.

### Statistical Analysis

Statistical significance was determined using student’s t-test. In all tests, the minimum level of significance for a 2-tailed test was set at p<0.01.

## Results

### Generation of MUC1 SP-specific Antibodies

**Figure 1:**
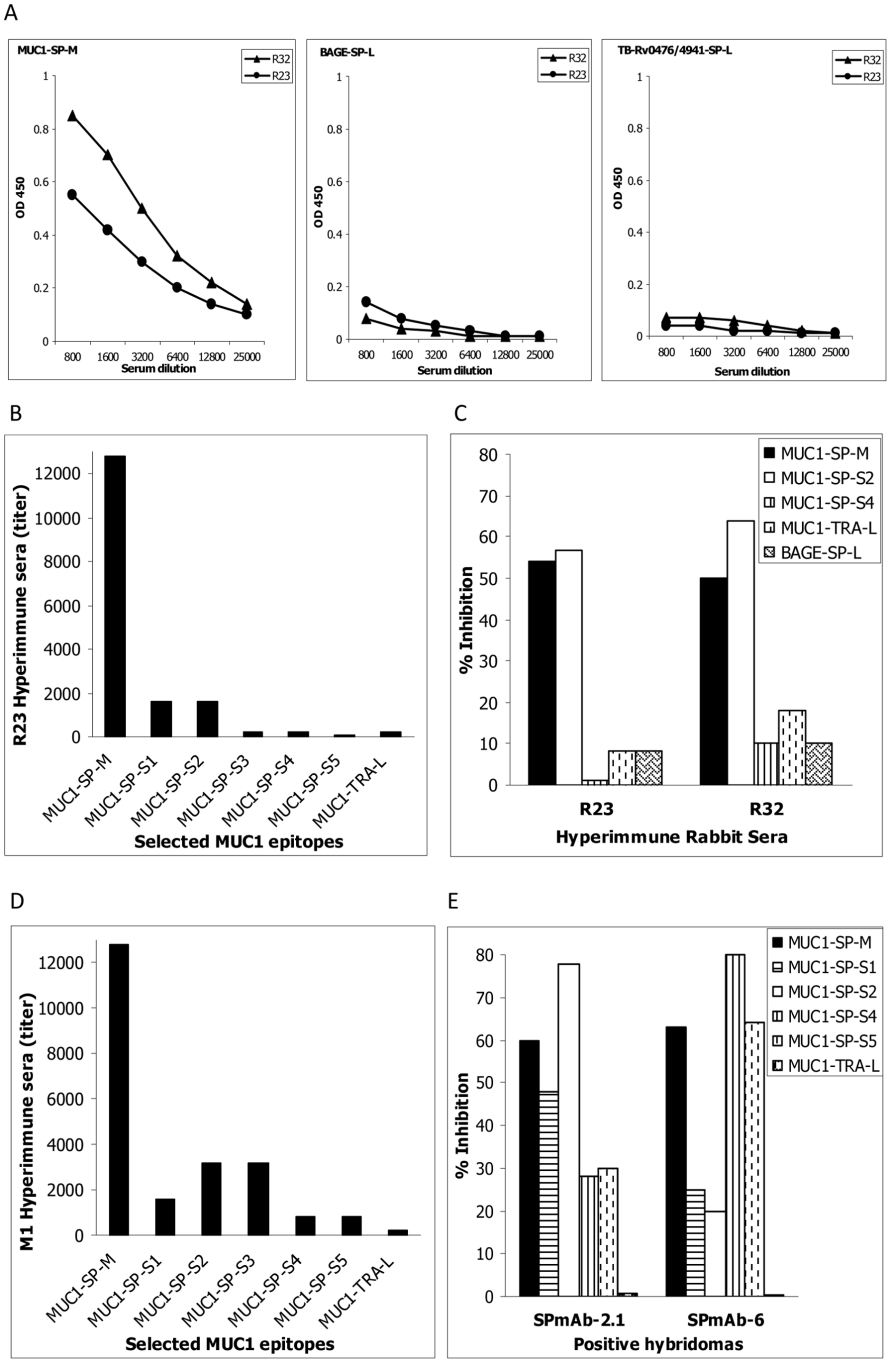
Generation of MUC1 SP-specific antibodies. The specificity and defined minimal epitope of the generated anti-MUC1 SP polyclonal antibodies (A–C) and mAbs SPmAb-2.1 and SPmAb-6 (D–E) were evaluated against the immunizing peptide MUC1-SP-M and its internal epitopes MUC1-SP-S1 to S5, in ELISA assays. MUC1's TRA epitope, MUC1-TRA-L, and the non-MUC1 SP domain BAGE-SP-L, were used in these assays as negative controls.

To promote exploration of the nature, specificity and location of the antigen leading to the generation of autoantibodies to MUC1-SP-L, the KLH-conjugated 17-mer MUC1-SP-M peptide (Table 1) was used to generate polyclonal and mAbs. MUC1-SP-M was chosen based on an in silico prediction for high density MHC Class II and B-cell epitopes and on the considerable concentration of autoantibodies against this peptide, found in the sera of MM patients [34]. Anti-MUC1-SP-M polyclonal antibodies (positive sera titer at ≤1∶25,000) were obtained in mice (data not presented) and in two immunized rabbits R23 and R32 (positive at titer ≤1∶12,800) (Table 1 and Fig. 1A left panel). The anti-MUC1 humoral response demonstrated limited cross reactivity (titres of <1∶800 dilutions) to both eukaryote (BAGE-SP-L, Table 1 and Fig. 1A middle panel) and prokaryote (TB-Rv0476/4941-SP-L, Table 1 and Fig. 1A right panel) SPs. The MUC1-SP-M inner epitopes most recognized by R23 were MUC1-SP-S1 and MUC1-SP-S2 (Table 1, Fig. 1B), both located at the MUC1 SP C-terminus. Competition assays evaluating the specificity of the polyclonal antibodies demonstrated >50% inhibition of both R23- and R32-derived antibodies by MUC1-SP-M and its inner epitope MUC1-SP-S2 (Fig. 1C). Inhibition of <10% was achieved by other MUC1 SP epitopes, in particular MUC1-SP-S4 and MUC1-TRA-L, and by the BAGE SP domain BAGE-SP-L. Based on these results, the minimal epitope of R23 and R32 is located within the MUC1-SP-S1 and MUC1-SP-S2 sequences, i.e., amino acids 12–21.

**Table 1:** Peptides used in this study.

Position	Indication/Target/Domain	Length	Sequence	Published ID	Internal ID^1^
1–21	Cancer/MUC1-SP	21-mer	MTPGTQSPFFLLLLLTVLTVV**-NH2**	MUC1-SP-L, VXL100^28^	MUC1-SP-L
10–21	Cancer/MUC1-SP	17-mer	**KK-**FLLLLLTVLTVV**-KKK**	MUC1-SP-M^34^	MUC1-SP-M
13–21	Cancer/MUC1-SP	9-mer	LLLTVLTVV	MUC1D6^21^	MUC1-SP-S1
12–20	Cancer/MUC1-SP	9-mer	LLLLTVLTV	MUC1C6^21^, M1.2^20^	MUC1-SP-S2
10–18	Cancer/MUC1-SP	9-mer	FLLLLLTVL	–	MUC1-SP-S3
5–13	Cancer/MUC1-SP	9-mer	TQSPFFLLL	MUC1-SP-S4^28^	MUC1-SP-S4
7–15	Cancer/MUC1-SP	9-mer	SPFFLLLLL	MUC1-SP-S5^28^	MUC1-SP-S5
130–154	Cancer/MUC1-TRA	25-mer	STAPPAHGVTSAPDTRPAPGSTAPP	MUC1-TRA-L^28^, BP25	MUC1-TRA-L
1–17	Cancer/BAGE-SP	17-mer	MAARAVFLALSAQLLQA	–	BAGE-SP-L
1–19	Mycobacterium tuberculosis Rv0476/4941-SP	19-mer	MLVLLVAVLVTAVYAFVHA**-NH2**	VXL211^31^ TB-Rv0476/4941-SP-L^34^	TB-Rv0476/4941-SP-L

^1^ Nomenclature used for internal ID is: target antigen (e.g. MUC1), targeted domain (e.g. SP or TRA), and peptide lengths were "S" (short), "M" (moderate), "L" (long)**.**

Following establishment of MUC1-SP-M immunogenicity in rabbits, we chose to generate mAb in mice. Sera collected from MUC1-SP-M-immunized mouse No. 1 (M1) (Fig. 1D) strongly bound the immunizing peptide, MUC1-SP-M (>1∶12,800 titer), moderately bound MUC1-SP-S1, MUC1-SP-S2 and MUC1-SP-S3 (>1∶1800–3600 titers) and weakly bound MUC1-SP-S4 and MUC1-SP-S5 (>1∶800 titer). The sera failed to bind MUC1-TRA-L. Binding experiments with sera from mouse No. 2 demonstrated the highest titers (>1∶1600) to peptides MUC1-SP-S4 and MUC1-SP-S5 (data not shown). The cloning efforts yielded two mAbs, one with an Ig-gamma1 isotype, designated SPmAb-2.1, and the second with an Ig-gamma2a isotype, designated SPmAb-6. mAb specificity was validated by binding and competition assays (Fig. 1E) with various free peptides (Table 1). MUC1-SP-S2 most potently inhibited SPmAb-2.1, while MUC1-SP-S4 most effectively inhibited SPmAb-6 binding, both defining the minimal epitopes of the respective antibodies.

### Anti-MUC1 SP Antibodies Bind to MUC1-positive Tumor Cells

Flow cytometry analysis demonstrated moderate-high SPmAb-2.1, SPmAb-6 and R23IgG binding to MUC1-expressing solid and haematological tumors (Table 2). The anti-MUC1 TRA mAb H23 [35], which served as a MUC1 positive control, showed unequivocal reactivity, with binding strength similar to that of the R23IgG antibodies. In contrast, MUC1-negative melanoma cell-lines, SK-mel-28 and SK-mel-1, and the MUC1-negative ovarian cell-line, ES-2, consistently failed to react (low geometric mean) with all tested antibodies (Table 2), demonstrating antibody selectivity to MUC1 SP. Further support of the tumor cell- and MUC1 SP-specificity of the antibodies, was provided by absence of binding of SPmAb-2.1, SPmAb-6 mAbs and R23IgG antibodies to human white blood cells, in particular, to CD3^+^ T-cells, CD20^+^ B-cells and CD14^+^ myeloid cells, as well as to naïve normal human mammary epithelial cells (HMEC) (Table 2).

**Table 2:** *The cellular* expression of MUC1 SP domain on MUC1 positive tumor cell-lines and primary naïve cells.

Human tumor Cell-lines Primary cells	Origin	Mouse control	H23	SPmAb-6	SPmAb-2.1	Rabbit control		R23IgG
		Fluorescence intensity (Geometric mean)						
ES-2	Ovarian Carcinoma	234	232	248	237	251	247	
OVCAR-3	Ovarian Carcinoma	370	4456	11440	1019	418	10800	
MCF7	Breast Carcinoma	230	1004	1091	413	236	4705	
MDA-453	Breast Carcinoma	182	580	559	290	165	2443	
MDA-231	Breast Carcinoma	245	966	591	312	215	1931	
Raji	B-Lymphoblastic Leukemia	131	303	307	179	128	519	
Ramos	B-Lymphocytic Leukemia	198	558	660	402	111	384	
U266	Multiple Myeloma	485	2039	661	574	471	1716	
RPMI 8226	Multiple Myeloma	745	2585	1190	1154	738	2331	
ARH-77	Plasma cell Leukemia	176	668	691	439	161	3966	
SK-mel-28	Melanoma	117	119	114	112	113	117	
SK-mel-1	Melanoma-Metastasis	352	325	342	345	350	348	
Naïve epithelial cells	Mammary epithelial cells (HMEC)	319	338	328	314	380	343	
		**Fluorescence intensity (% Positive staining)** 1						
T-Lymphocytes (CD3+)	White blood cells	0	0.135	0.062	0.097	0.043	0.038	
B-Lymphocytes (CD20+)	White blood cells	0	0.031	0.003	0.009	0.003	0.41	
Myeloid (CD14+)	White blood cells	0.003	0.463	0.325	0.23	0.026	0.032	

^1^% of positive staining was applies in cell which undergo double staining.

### Anti-MUC1 SP Antibodies Localize to the Membranes of MUC1-positive Tumor Cells

**Figure 2:**
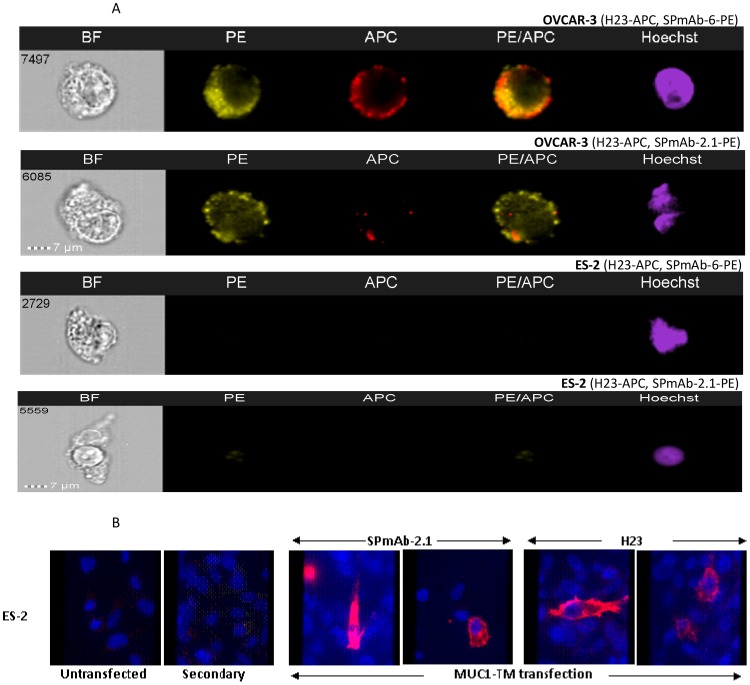
Detection of MUC1 SP on the membrane of MUC1-positive tumor cells. The cell surface presence and similarity analysis of the MUC1 SP domain and MUC1 TRA was evaluated on OVACAR-3 MUC1-positive and ES-2 MUC1-negative tumor cell lines by imagestream analysis using PE- and APC-tagged anti-MUC1 SP mAbs SPmAb-2.1, SPmAb-6, and anti-MUC1 TRA H23 mAb (A). Fluorescence microscopy analysis was also performed on ES-2 MUC1-transfected ovarian cells vs. the parent MUC1-negative cells (B). BF stand for bright field and secondary stand for anti-mouse IgG antibodies.

The localization of the antigen recognized by the different MUC1 SP antibodies was determined via imagestream analysis. MUC1 SP presence was observed, using PE-conjugated SPmAb-2.1, SPmAb-6 mAb and APC-conjugated H23 MUC1 TRA mAb, on the cell surface of MUC1-positive ovarian cell line OVCAR-3, while no expression was observed on the MUC1-negative ovarian cell line ES-2 (Fig. 2A). The merged images of 30000 cells indicate that MUC1 SP and MUC1 TRA localize mainly alone on the membrane of the cells. MUC1 SP was observed as an independent membranal domain, as demonstrated by a single PE^bright^ membranal staining, in 47% and 56% of the analyzed cells, upon treatment with SPmAb-6 and SPmAb-2.1, respectively. Colocalization of the MUC1 SP and MUC1 TRA molecules, demonstrated by a double PE^bright^/APC^bright^ membranal staining, was minimal, and observed in 18% and 27% of the cells exposed to SPmAb-6 and SPmAb-2.1, respectively (data not shown). A high similarity between MUC1 SP and MUC1 TRA staining (similarity coefficients >1.0) was observed (data not shown), indicating colocalization of these molecules on very few selected cells. Additional confirmation of the MUC1-specificity of these antibodies was obtained by specific binding of the anti-MUC1 SP mAb SPmAb-2.1 and the MUC1 TRA mAb H23 to ES-2 MUC1-negative ovarian cells only upon their transfection with the MUC1-TM expression construct (Figure 2B).

### Anti-MUC1 SP Antibodies Bind MUC1-expressing MM Plasma Cells in Fresh Bone Marrow Aspirates

**Figure 3:**
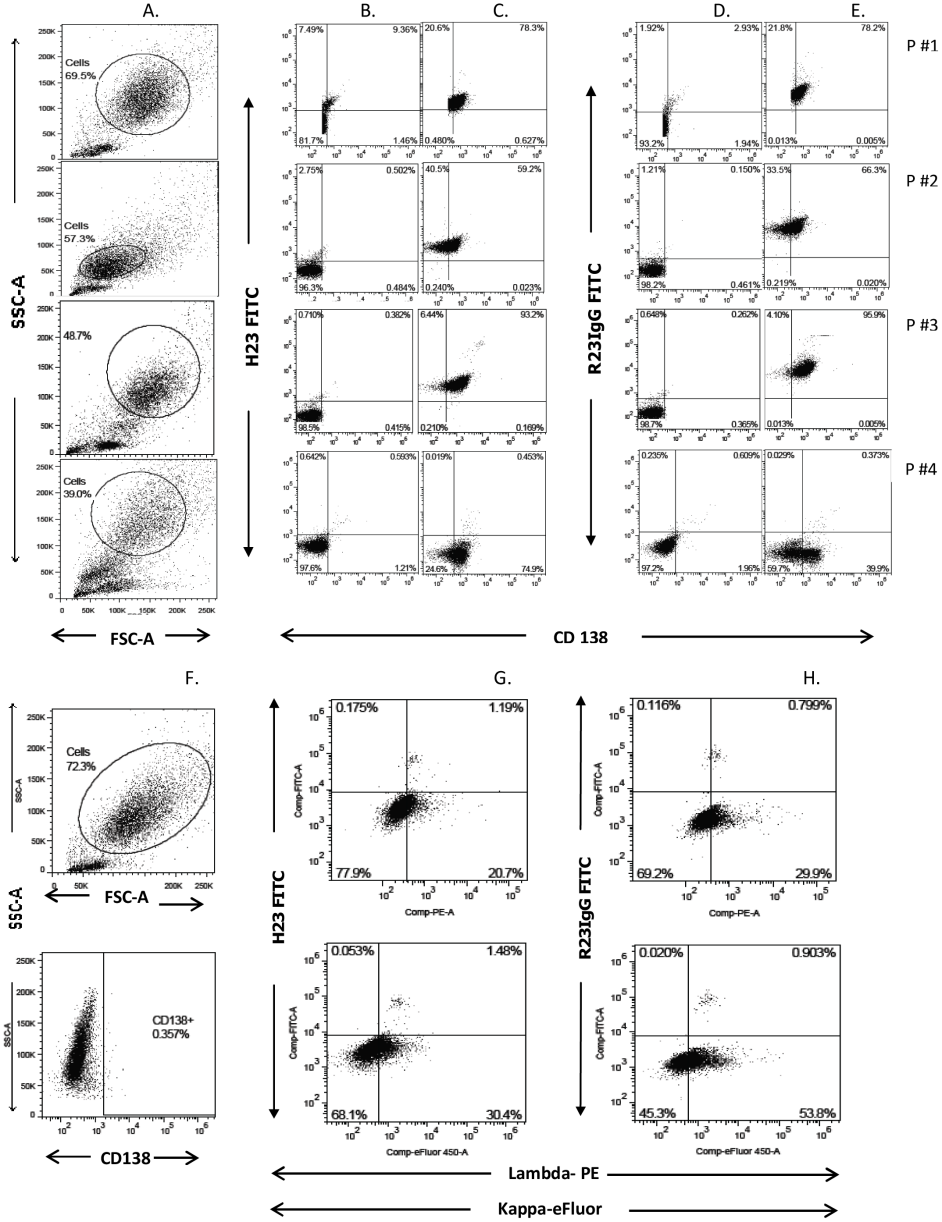
Anti-MUC1 SP antibodies bind MUC1-expressing MM cells in fresh human BM aspirates. The cell surface presence of the MUC1 SP domain was evaluated by gated FACS analysis on PC cells in fresh BM aspirates obtained from MM patients (A–E) and normal naïve sample (F–H). Polyclonal anti-MUC1 SP (R23IgG) was used to determine MUC1 SP expression; MUC1 TRA domain expression was determined with H23 mAb. For each experiment, species-matched control antibodies for MUC1 staining were used: either normal mouse IgG–FITC (B), or normal rabbit IgG-FITC (D). Anti-MUC1 SP mAb (SPmAb-2.1) was used to determined MUC1 SP expression; MUC1 TRA domain expression was determined with H23 mAb.

Freshly obtained bone marrow (BM) aspirates of four MM patients were used to investigate whether R23IgG antibodies selectively bind primary tumor cells in an ex-vivo, heterogenous setting. Suspected plasma cells (PC) were gated (Fig. 3 column A) and their phenotype was verified by staining for kappa light and lambda chains (data not shown). The gated population was next analyzed for expression of MUC1 TRA (Fig. 3 column C) and MUC1 SP (Fig. 3 column E) on CD138-positive cells. Species-matched control antibodies for MUC1 staining were used for each experiment (Fig. 3 columns B and D). The CD138-positive PC of BM aspirates of MM patients #1, #2 and #3 bound R23IgG (78.2%, 66.3%, 95.9%, respectively, of the analyzed cell population) and H23 (78.3%, 59.2%, 93.2%, respectively, of the analyzed cell population) (Fig. 3 columns C and E). In contrast, the fourth aspirate (P#4) demonstrated low reactivity to both H23 and R23IgG (0.37%, and 0.45%, respectively, of the analyzed population), despite moderate CD138 expression (74.9% and 39.9% of cells, respectively) in this aspirate. As a control, we ran a similar flow cytometry analysis on a BM aspirate derived from a naïve, healthy donor. Cells in this analysis were gated for kappa and lambda expression, as CD138 expression was negative (Fig. 3 columns F). Results (Fig. 3 columns G and H) confirmed minimal binding of ∼1% for both MUC1-TRA and MUC1 SP on both kappa and lambda chains. In summary, these findings demonstrate that R23IgG and H23 specifically recognize malignant PC, rendering MUC1 SP an antigen suitable for treatment selection and monitoring purposes.

### Compliment-dependent Cytotoxicity Mediated by Anti-MUC1 SP Antibodies

**Figure 4:**
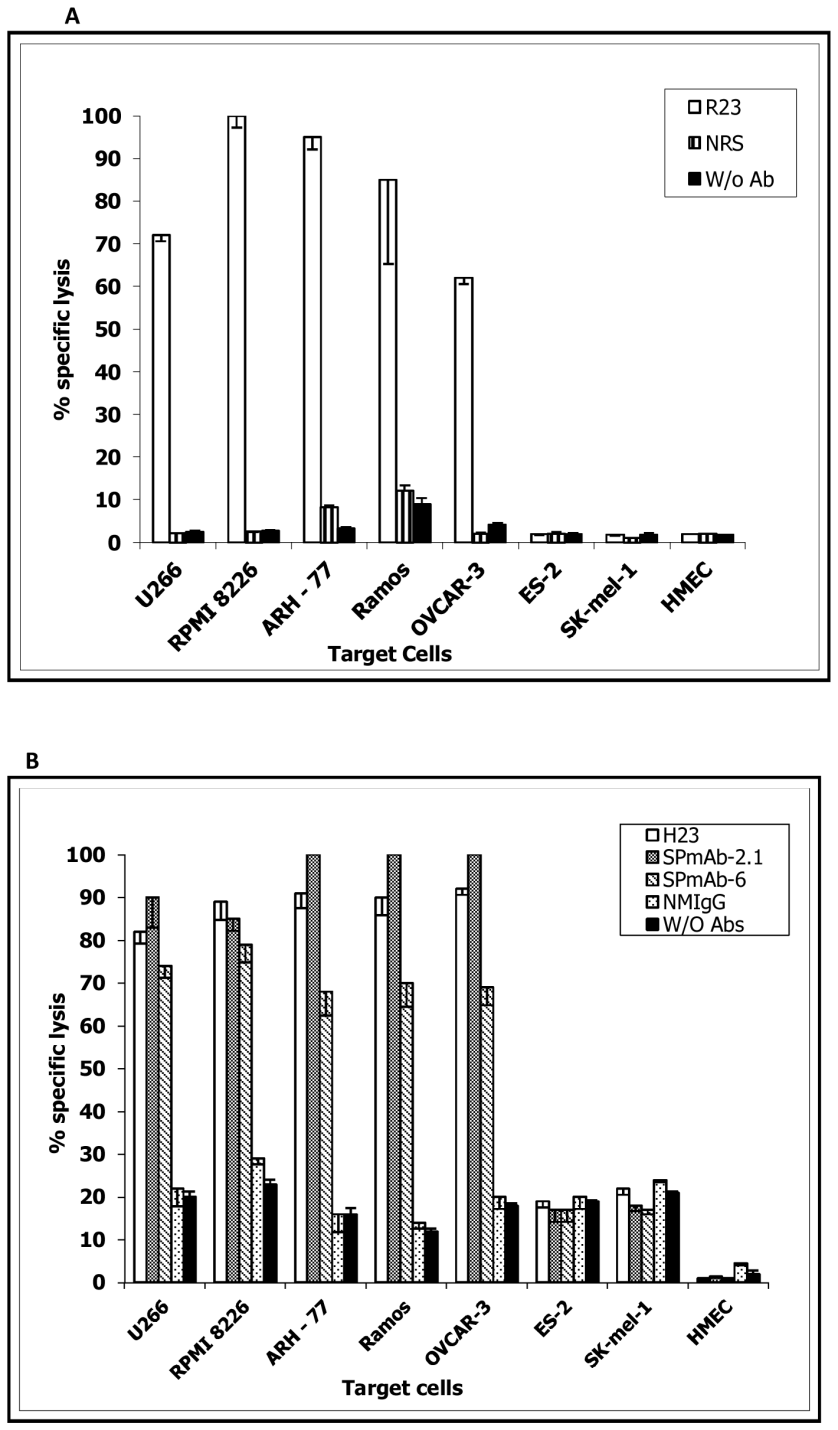
Efficacy and specificity of anti-MUC1 SP antibodies–mediated CDC mediated. The cytotoxic properties of the anti-MUC1 SP polyclonal R23IgG (A) and monoclonal SPmAb-2.1, SPmAb-6 mAb (B) were evaluated by CDC analysis using various solid, non-solid, MUC1-positive and -negative tumors and HMEC. The MUC1 TRA domain-specific mAb H23, NMS and NRS and no (W/O) antibodies were evaluated as controls.

The translatability of antigen and tumor specificities of anti-MUC1 SP antibodies to functional immunotherapeutic potential was demonstrated by effective lysis of MUC1-expressing cells by R23IgG (Fig. 4A), SPmAb-2.1 and SPmAb-6 (Fig. 4B), indicating their potential as anti-tumor effectors. R23IgG antibodies mediated 60–100% lysis of solid OVACAR-3 ovarian cells, and of hematological, U266, RPMI 8226, MM, ARH-77 and Ramos Leukemia tumor cells. In a similar manner, SPmAb-2.1 and SPmAb-6 triggered highly specific lysis of >90% and 60–80%, respectively, of the same cell lines. In comparison, H23 induced lysis of 80–90% of the tested cell populations (Fig. 4B). Lysis induced by all anti-MUC1 antibodies was highly significant (p<0.001 in Student's t-test) in MUC1-expressing tumor cell lines, when compared to the ovarian cell line ES-2, the melanoma cell line SK-mel-1 and NHEC, three MUC1-negative cell lines. Generally, CDC lysis efficacy strongly correlated with MUC1 cell surface expression levels, evaluated by flow cytometry analysis (table 2), with the exception of SPmAb-6 mAb, which demonstrated high cell surface presence but moderate CDC.

## Discussion

Despite extensive research concerning the immunotherapeutic potential of MUC1, there is still no licensed product against this target. Failure to isolate cell-bound, insoluble MUC1 epitopes has hindered development of an effective MUC1-related immunotherapeutic agent. Significant advance was made by Rubinstein and colleagues [37] and more recently, by Pichinuk and colleagues [24], who produced antibodies against the alpha/beta junction of cellular MUC1s. These antibodies showed highly selective MUC1 binding and induced cytotoxicity of MUC1-positive tumors, when linked to a toxin [24]. Based on our previous observations of naturally generated anti-MUC1 SP autoantibodies in MM patients, but not in naïve healthy donors, [34] the current study adopted a different strategy to target the insoluble MUC1 SP domain. The R23IgG, SPmAb-2.1 and SPmAb-6 antibodies we raised, specifically bound MUC1 SP, without binding unrelated SPs (Fig. 1A) and MUC1-TRA epitopes (Fig. 1B–E). The minimal epitopes recognized by these antibodies were located in the carboxy-terminal of MUC1 SP (Fig. 1C and 1E).

Past observations suggests that SP domains could potentially direct proteins to either the cell membrane or the extracellular compartment [33]. Uncleaved SP domains, as in the case of protease inhibitors, such as plasminogen activator inhibitor-2 [38], or its chick homologue, ovalbumin [39], guide the protein to the ER and fails to support membrane docking, yielding secretion of the intact protein. In the case of the lymphocytic choriomeningitis virus (LCMV) precursor glycoprotein C, insertion of the protein into the ER membrane is mediated by an unusual 58 amino acid-long SP bearing an extended N-terminal region. Although the SP is cleaved off by SPase, it remains noncovalently attached to the glycoprotein without being processed by SPP. This mechanism has been implicated in cell surface expression of the SP with the entire glycoprotein complex [40]. Our current findings (Table 2, Fig. 2) demonstrate for the first time, tumor-associated cell surface presence of MUC1 SP on several human tumor cell lines and primary tumors, with negligible presence on HMEC and white blood cells.

Unpredictably, our observations (Fig. 2A, B) suggest that MUC1 SP can migrate to the cell surface, primarily as an independent entity disassociated with the full MUC1-TM, but also as part of the full MUC1-TM isoform. A number of plausible mechanisms lie at the basis of these findings:

1. Recognition of cell-bound SP as an independent entity could result from malfunctioning, cancer-associated SPP, which normally cleaves the SP molecule inside the ER membrane and releases its carboxy-terminal into the ER lumen. Cancer-associated failure in SPP activity, could plausibly lead to the vesicular transport of SP, with part of the ER membrane, to the cell membrane, exposing its carboxy-terminal to the immune system. This mechanism can better explain the preferred recognition of MUC1 SP's carboxy-terminal by the generated anti-SP antibodies.
2. Recognition of cell-bound MUC1-TM-associated SP could result from non-covalent binding of the SP to the MUC1-TM following cleavage by SPase, which normally cleaves the SP domain from the rest of the protein in the ER. This mechanism was previously described for the LCMV [40], but not for the TAA-derived SP.
3. Recognition of cell-bound MUC1-TM-associated SP could also result from improper function of the cancer-associated, ER–associated SPase. Cancer-associated mutations in SPase have not been described. Alternatively, this phenomenon can be linked to inability of the SPase to cleave all the translated MUC1 molecules which are overproduced in the transformed tumor cell.

Since the last two suggested mechanisms would lead to increased levels of SPase non-cleaved MUC1 SP, bound to the cell surface with the alfa subunit of the MUC1-TM, MUC1 SP should be released to the blood either alone or as part of the cleaved alpha subunit. Since soluble MUC1 SP, was neither detected in patient sera [34] nor in the concentrated supernatant of MUC1-positive tumour cell lines (unpublished observations), we suggest that: (a) As shown (figure 2A) these mechanisms are responsible for only a small fraction of the surface-bound MUC1 SP domain which cannot be detected by our assay, and/or, (b) the MUC1 SP released to the blood is immediately degraded by serum proteases.

It is most probable that a combination of more than one of the proposed mechanisms leads to the strong yet specific, membranal MUC1 SP presence detected via staining intensity of tumor cell lines and patient-derived PC with the R23IgG antibodies. This intensity closely resembled the intensity of staining observed with the anti-MUC1 TRA domain mAb H23, although the later has multiple epitopes on each molecule, which lead to a strong signal even in lower antigen concentration.

Unloaded antibodies against membranal TAA are seldom curative when delivered as monotherapy, and usually facilitate antibody-dependent, cell-mediated cytotoxicity and/or CDC [41]. As an initial demonstration of the clinical applicability of anti-SP antibodies, we evaluated the capacity of anti-MUC1 SP antibodies to mediate CDC in-vitro. Our results (Fig. 4) confirm that anti-MUC1 SP antibodies not only bind MUC1-positive tumor cells and MM patient-derived PC, but also effectively mediate CDC of both solid and haematological MUC1-positive tumor cells lines, but not of NHEC. Lysis failure, at least in the case of SK-mel-1, was not related to CDC resistance, as these cells were previously shown to be sensitive to CDC [42]. In light of the results presented in this study, including the demonstrated tumor specificity of MUC1 SP, we propose further efforts toward targeting cell-bound MUC1-positive tumors using anti-MUC1 SP antibodies, specific to the cell-bound, rather than the soluble MUC1.

Success of anti-cancer products relies heavily on proper patient selection, especially in those with minimal residual disease. Yet, at least in MM patients, vast heterogeneity in such flow cytometry-based analysis is observed [43]. Identification of patient populations expressing the epitopes targeted by a specific drug can thereby significantly promote therapeutic success. FACS analysis with R23IgG, of freshly aspirated MM patient-derived samples (Fig. 3), clearly implied that these antibodies can serve as a selection tool prior to and potentially during MUC1-directed therapy. Anti-MUC1 SP antibodies specifically bind MUC1-expressing tumor cells in an unmanipulated, heterogeneous cell population, with minimal nonspecific binding to naïve BM cells. In addition, R23IgG reactivity in the aspirates was mostly concordant with immunoreactivity of anti-CD138 (Syndecan-1), a well-established PC marker [44] (Fig. 3). A similar selection analysis of PC from BM aspirate of MM patients, based upon kappa/lambda and CD138 expression, was recently described by Nakayama et al [45] and confirmed the rationale of our analysis. The analysis also revealed positive MUC1 expression in a small population of CD138-negative cells. This finding is logical since a small population of CD138-negative PC were previously characterised and were associated with more advanced malignancy [46].

The ultimate objective of an anti-MUC1 therapeutic vaccine is to induce antigen-specific cellular and humoral response. Our earlier findings, regarding the promiscuous MHC binding of the MUC1 SP domain and, consequently, its ability to induce a broader T-cell response [28], together with the present results, showing cell-bound MUC1 SP, implies that targeting MUC1 SP-presenting tumors with the ImMucin vaccine, may lead to the desired activation of MUC1-specific CD4+, CD8+ T-cell and antibodies. The in vivo targeting capacities of anti-SP antibodies and the translation of these antibodies to other TAAs, remain to be determined.
